# The burden and trend of gastric cancer and possible risk factors in five Asian countries from 1990 to 2019

**DOI:** 10.1038/s41598-022-10014-4

**Published:** 2022-04-08

**Authors:** Fei-Long Ning, Jun Lyu, Jun-Peng Pei, Wan-Jie Gu, Nan-Nan Zhang, Shi-Yi Cao, Yong-Ji Zeng, Masanobu Abe, Kazuhiro Nishiyama, Chun-Dong Zhang

**Affiliations:** 1 Department of General Surgery, The Affiliated Xuzhou Hospital of Nanjing University of Chinese Medicine, Xuzhou Hospital of Traditional Chinese Medicine, Xuzhou, 221003 China; 2 Department of Clinical Research, The First Affiliated Hospital of Jinan University, Guangzhou, 510630 China; 3 Department of Gastrointestinal Surgery, The Fourth Affiliated Hospital of China Medical University, No.4 Chongshan East Road, Huanggu, Shenyang, 110032 China; 4 Evidence-Based Medicine Group, Department of Anesthesiology, Nanjing Drum Tower Hospital, The Affiliated Hospital of Nanjing University Medical School, Nanjing, 210008 China; 5 State Key Laboratory of Cancer Biology and National Clinical Research Center for Digestive Diseases, Xijing Hospital of Digestive Diseases, Fourth Military Medical University, Xi’an, 710032 China; 6 School of Public Health, Tongji Medical College, Huazhong University of Science and Technology, Wuhan, 430030 China; 7 Section of Gastroenterology, Department of Medicine, Baylor College of Medicine, Houston, TX 77030 USA; 8 Division for Health Service Promotion, The University of Tokyo, Tokyo, 113-8655 Japan; 9 Department of Gastrointestinal Surgery, Graduate School of Medicine, Kyoto University, Kyoto, 606-8507 Japan

**Keywords:** Gastroenterology, Risk factors, Cancer

## Abstract

The burdens and trends of gastric cancer are poorly understood, especially in high-prevalence countries. Based on the Global Burden of Disease Study 2019, we analyzed the incidence, death, and possible risk factors of gastric cancer in five Asian countries, in relation to year, age, sex, and sociodemographic index. The annual percentage change was calculated to estimate the trends in age-standardized incidence rate (ASIR) and age-standardized death rate (ASDR). The highest ASIR per 100,000 person-years in 2019 was in Mongolia [44 (95% uncertainty interval (UI), 34 to 55)], while the lowest was in the Democratic People’s Republic of Korea (DPRK) [23 (95% UI, 19 to 29)]. The highest ASDR per 100,000 person-years was in Mongolia [46 (95% UI, 37 to 57)], while the lowest was in Japan [14 (95% UI, 12 to 15)]. Despite the increase in the absolute number of cases and deaths from 1990 to 2019, the ASIRs and ASDRs in all five countries decreased with time and improved sociodemographic index but increased with age. Smoking and a high-sodium diet were two possible risk factors for gastric cancer. In 2019, the proportion of age-standardized disability-adjusted life-years attributable to smoking was highest in Japan [23% (95% UI, 19 to 28%)], and the proportions attributable to a high-sodium diet were highest in China [8.8% (95% UI, 0.21 to 33%)], DPRK, and the Republic of Korea. There are substantial variations in the incidence and death of gastric cancer in the five studied Asian countries. This study may be crucial in helping policymakers to make better decisions and allocate appropriate resources.

## Introduction

Over one million new cases of gastric cancer and 769,000 deaths were estimated in 2020 worldwide, with the highest incidence rates in several Asian countries, especially Japan, Mongolia, and China^1^. Although the incidence rate of gastric cancer has been declining, that among young adults has been increasing rapidly during the past several decades^2–5^. Efforts to estimate the burden of gastric cancer remain a formidable challenge, especially in high-prevalence countries in Asia. The epidemiology of gastric cancer shows substantial geographical heterogeneity, and its incidence varies widely between high- and low-risk countries^6^. The reason for this geographical variation remains elusive, but may correlate with the incidences of *Helicobacter pylori* infection, smoking, and the consumption of salt and salt-preserved foods^7–9^.

Gastric cancer is common, especially in Asian countries, including Japan, China, Mongolia, and the Republic of Korea. For example, China accounts for half of all cases of gastric cancer worldwide, while it remains the most common type of cancer among men in Japan. However, the latest data on the burdens and trends in incidence and death rates of gastric cancer remain poorly understood, especially in terms of disability-adjusted life-years (DALYs) attributable to possible risk factors of gastric cancer in those countries. A better understanding of the burdens and trends are therefore needed to develop effective strategies for the prevention and early detection of gastric cancer, to extend patients’ life expectancy^10,11^. This study therefore aimed to analyze the burdens and trends of gastric cancer incidence and death, and the effects of possible risk factors on DALYs worldwide and in five Asian countries, including Mongolia, China, the Republic of Korea, Japan, and the Democratic People’s Republic of Korea (DPRK), from 1990 to 2019, in relation to year, age, sex, and sociodemographic index (SDI), based on the Global Burden of Disease (GBD) Study 2019.

## Materials and methods

### Data sources

The data source and statistical methods for GBD 2019 have been described elsewhere^12,13^. Briefly, the GBD estimated data for 369 diseases and injuries and 84 risk factors at global, regional, and national levels from 1990 to 2019. We extracted data from the Global Health Data Exchange (GHDx) website (http://ghdx.healthdata.org/gbd-results-tool). We used the term “stomach cancer” as the “cause”; we used the terms “incidence” and “death” as the “measure”.

Gastric cancer included all diagnoses coded according to the International Classification of Disease 10th Revision, ICD-10 as C16–C16.9, Z12.0, and Z85.02–Z85.028^12^. Data on the age-standardized incidence rate (ASIR) and age-standardized death rate (ASDR) at global and national levels were extracted from GBD 2019^14^. The geographic locations included Mongolia, China, the Republic of Korea, Japan, and DPRK. Estimates were reported with 95% uncertainty intervals (UIs).

### Estimates of annual percentage change (APC)

APC was estimated to test time trends in ASIRs and ASDRs from 1990 to 2019 using Joinpoint regression analysis (Joinpoint Regression Program, Version 4.7.0.0; National Cancer Institute, Calverton, MD, USA)^15^. We estimated the time trend by APC with 95% confidence intervals (CIs) for each segment identified by the model, and tested which trends between joinpoints were significantly different from zero. A *P* value < 0.05 was considered statistically significant. The ASIR or ASDR was considered as an increasing trend if both the APC value and the lower 95% CI were > 0, and a decreasing trend if both the APC value and the upper 95% CI were < 0.

Furthermore, a “stable” trend was characterized as a change ≤ 0.5% (− 0.5% ≤ APC ≤ 0.5%) and a non-significant APC; a “non-significant change” trend was characterized as a change > 0.5% (APC <  − 0.5% or APC > 0.5%) with a non-significant APC; a “rising” trend was characterized as a significant APC > 0; and a “falling” trend was characterized as a significant APC < 0 (https://progressreport.cancer.gov/methodology).

### SDI

The SDI represents a composite index consisting of income per capita, average educational attainment, and total fertility rates^14^, ranging from the worst (0) to the best (1), reflecting the socio-development status. We further investigated the associations between SDI values and ASIR and ASDR in the five countries.

### Possible risk factors

Informative data on possible risk factors were extracted from the GHDx. In GBD 2019, one dietary factor (high-sodium diet) and one behavioral factor (smoking) relating to gastric cancer were reported in relation to sex, age, country, and year. The GBD 2019 established a comparative risk assessment (CRA) to estimate the DALYs attributable to possible risk factors. The CRA consisted of six key steps: (1) determining the inclusion of each risk-outcome pair; (2) estimating relative risk as a function of exposure for each risk-outcome pair; (3) estimating the distribution of exposure for each risk by age-sex-location-year; (4) determining the level of exposure with a theoretical minimum risk exposure level; (5) estimating the population attributable fraction (PAF) and burden; and (6) estimating the PAF and burden for combinations of risk factors^13^. DALYs were multiplied by the population attributable fraction (proportion by which DALYs would be decreased in a specific year if the exposure to a risk factor in the past was equal to the theoretical minimum risk exposure level) for each risk-outcome pair for a given age-sex-location-year^13^.

### Ethics approval and consent to participate

This study was approved by the Institute Ethics Committees of The Fourth Affiliated Hospital of China Medical University (EC-2021-KS-068) and was performed according to the guidelines of the Declaration of Helsinki. The data released from the Global Health Data Exchange query did not require informed consent.

## Results

### Incidence and death in 2019

**Figure 1:**
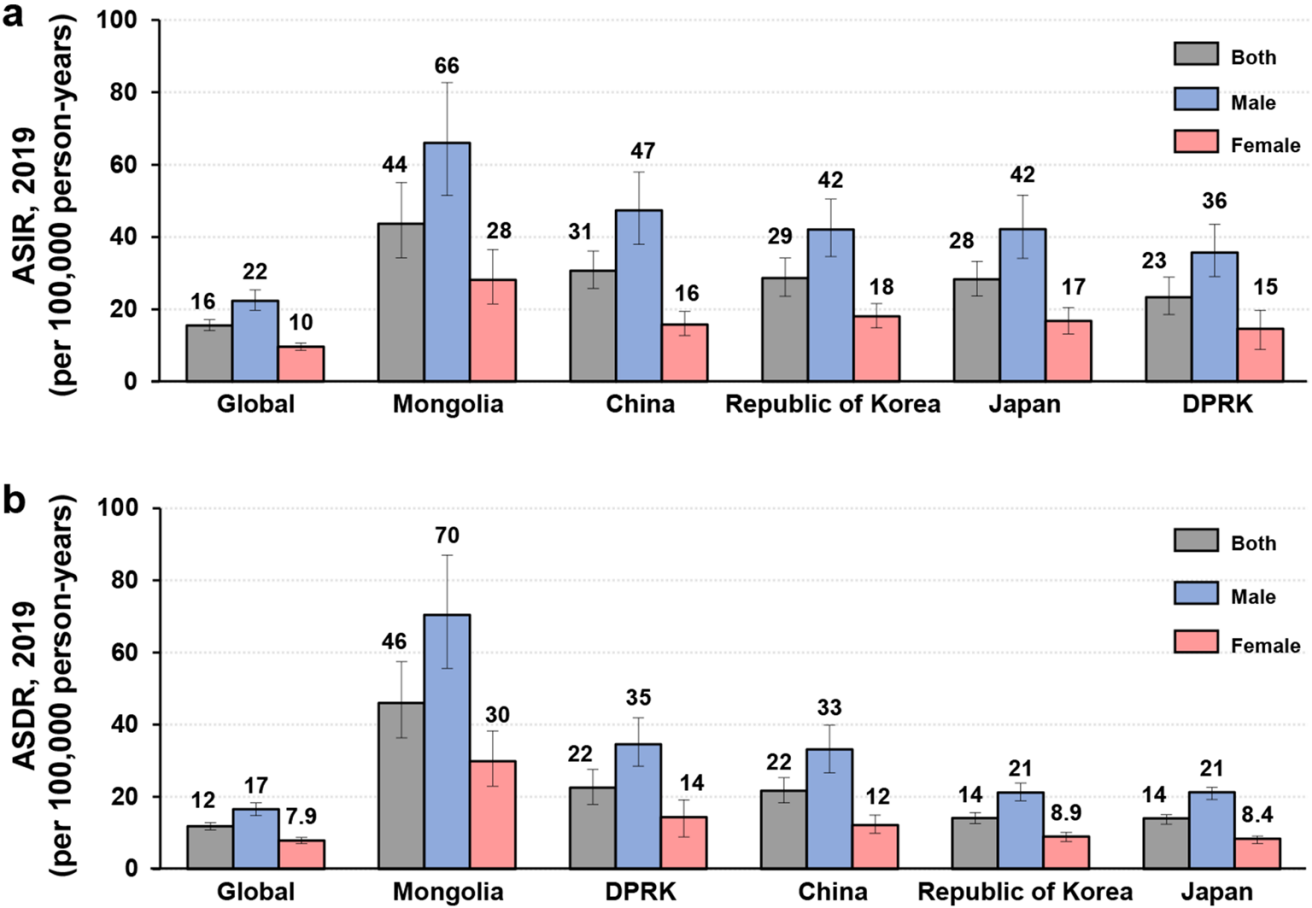
The ASIRs (**a**) and ASDRs (**b**) of gastric cancer worldwide and in five Asian countries in 2019 according to sex. ASDR, age-standardized death rate. ASIR, age-standardized incidence rate. Error bars indicate 95% uncertainty intervals.

The number of incident cases worldwide in 2019 was 1,269,806 (ASIR, 16 per 100,000 person-years) and the number of deaths was 957,185 (ASDR, 12 per 100,000 person-years) (Fig. 1a, b; Table 1). The ASIR per 100,000 person-years in 2019 was highest in Mongolia [44 (95% UI, 34 to 55)], followed by China [31 (95% UI, 26 to 36)], the Republic of Korea [29 (95% UI, 24 to 34)], and Japan [28 (95% UI, 24 to 33)], and lowest in the DPRK [23 (95% UI, 19 to 29)] (Fig. 1a; Table 1). The ASDR per 100,000 person-years was also highest in Mongolia [46 (95% UI, 37 to 57)], followed by the DPRK [22 (95% UI, 18 to 28)], China [22 (95% UI, 18 to 25)], and the Republic of Korea [14 (95% UI, 13 to 16)], and lowest in Japan [14 (95% UI, 12 to 15)] (Fig. 1b; Table 1). There were 421,539 deaths in China (95% UI, 353,520 to 493,176), which accounted for 44% of global deaths. The ASIRs and ASDRs were higher among males than females in all five countries.

**Table 1:** Incidence and deaths with percentage changes of gastric cancer for both sexes, between 1990 and 2019.

Location	Year 1990		Year 2019		Between 1990 and 2019	
	Incidence cases ASIR (95% UI)	Death cases ASDR (95% UI)	Incidence cases ASIR (95% UI)	Death cases ASDR (95% UI)	Percentage change, ASIR (95% UI)	Percentage change, ASDR (95% UI)
Global	883,396 (834,237 to 929,174) 22 (21 to 24)	788,317 (742,787 to 833,999) 20 (19 to 22)	1,269,806 (1,150,487 to 1,399,817) 16 (14 to 17)	957,185 (870,949 to 1,034,646) 12 (11 to 13)	–31% (–37% to –23%)	–42% (–47% to –36%)
China	317,335 (277,900 to 359,322) 38 (33 to 42)	305,467 (267,210 to 345,402) 38 (33 to 42)	612,821 (512,997 to 728,891) 31 (26 to 36)	421,539 (353,520 to 493,176) 22 (18 to 25)	–18% (–33% to 0.80%)	–42% (–53% to –30%)
DPRK	5,213 (3,947 to 6,631) 31 (24 to 39)	4,950 (3,778 to 6,282) 31 (24 to 38)	7,584 (5,987 to 9,409) 23 (19 to 29)	7,204 (5,694 to 8,907) 22 (18 to 28)	–25% (–42% to –2.0%)	–27% (–43% to –6.2%)
Japan	103,436 (99,545 to 105,881) 61 (59 to 63)	53,314 (50,875 to 54,577) 32 (31 to 33)	102,235 (83,884 to 120,371) 28 (24 to 33)	57,162 (48,002 to 62,076) 14 (12 to 15)	–54% (–61% to –46%)	–56% (–60% to –54%)
Mongolia	676 (563 to 807) 65 (55 to 77)	691 (580 to 821) 69 (58 to 81)	984 (755 to 1,263) 44 (34 to 55)	972 (747 to 1,240) 46 (37 to 57)	–33% (–49% to –13%)	–33% (–48% to –14%)
Republic of Korea	19,679 (18,765 to 20,540) 62 (59 to 64)	15,885 (15,179 to 16,573) 52 (50 to 55)	25,074 (20,656 to 29,936) 29 (24 to 34)	12,250 (10,962 to 13,503) 14 (13 to 16)	–53% (–61% to –44%)	–73% (–76% to –70%)

*ASDR* age-standardized death rate per 100,000 person-years, *ASIR* age-standardized death rate per 100,000 person-years, *DPRK* Democratic People’s Republic of Korea, *UI* uncertainty interval.

### Changes in incidence and death from gastric cancer between 1990 and 2019

**Figure 2:**
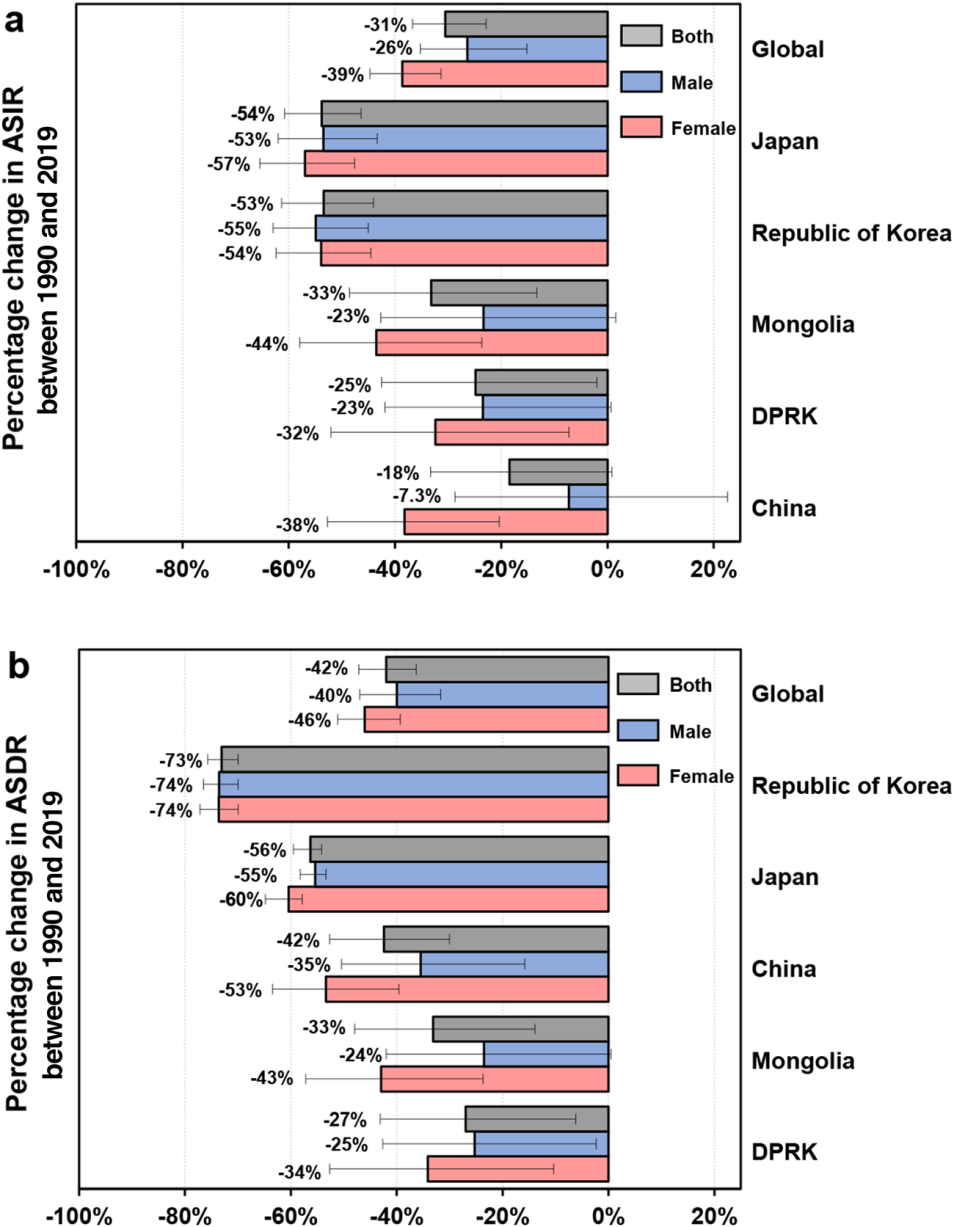
Percentage changes in ASIR (**a**) and ASDR (**b**) of gastric cancer worldwide and in five Asian countries between 1990 and 2019 according to sex. ASDR, age-standardized death rate. ASIR, age-standardized incidence rate. Error bars indicate 95% uncertainty intervals.

The global change in ASIR between 1990 and 2019 was − 31% and the change in ASDR was − 42% (Fig. 2a, b; Table 1). There were obvious decreases in the ASIR and ASDR between 1990 and 2019 in all five Asian countries (Fig. 2; Table 1). The greatest decrease in ASIR was in Japan [− 54% (95% UI, − 61 to − 46%)], followed by the Republic of Korea [53% (95% UI, − 61 to − 44%)], Mongolia [− 33% (95% UI, − 49 to − 13%)], the DPRK [− 25% (95% UI, − 42 to − 2.0%)], and China [− 18% (95% UI, − 33 to 0.80%)] (Fig. 2a, Table 1). The greatest decrease in ASDR was in the Republic of Korea [− 73% (95% UI, − 76 to − 70%)], followed by Japan [− 56% (95% UI, − 60 to − 54%)], China [− 42% (95% UI, − 53 to − 30%)], Mongolia [− 33% (95% UI, − 48 to − 14%)] and the DPRK [− 27% (95% UI, − 43 to − 6.2%)] (Fig. 2b, Table 1). Decreases in ASIR and ASDR were higher among females than males in all countries except the Republic of Korea.

**Figure 3:**
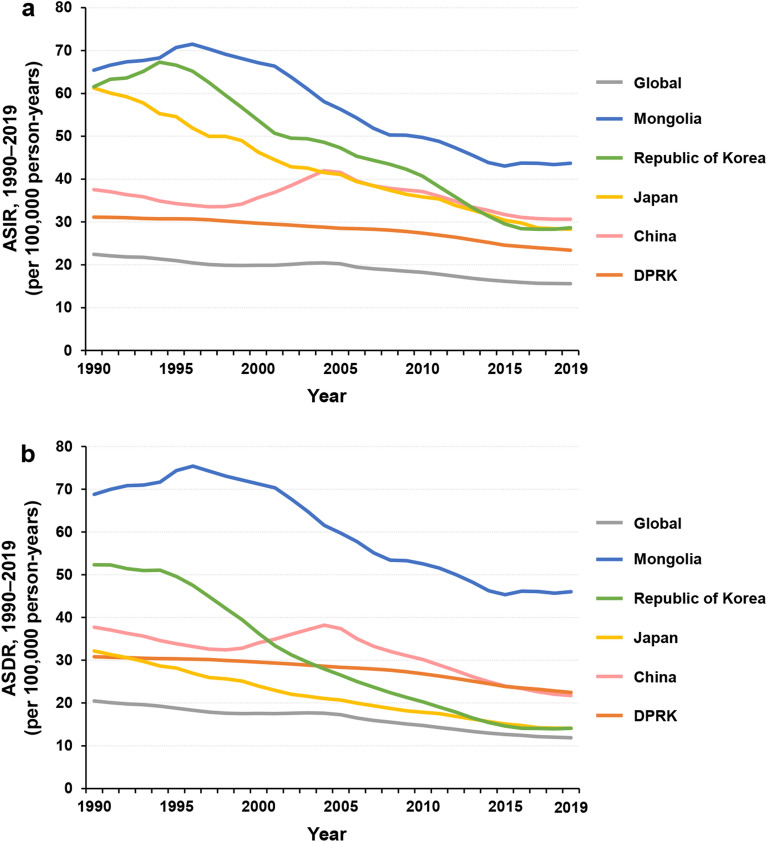
Time trends of ASIRs (**a**) and ASDRs (**b**) of gastric cancer worldwide and in five Asian countries from 1990 to 2019. ASDR, age-standardized death rate. ASIR, age-standardized incidence rate.

The greatest decrease in ASIR per 100,000 person-years was found in Japan, from 61 (95% UI, 59 to 63) in 1990 to 28 (95% UI, 24 to 33) in 2019 (Fig. 3a, Table 1). The Republic of Korea initially showed an increasing trend in ASIR from 1990 to 1994, but this then decreased continuously down to 29 (95% UI, 24 to 34) per 100,000 person-years in 2019. The ASIR in Mongolia initially increased and peaked in 1996, and then decreased continuously to 44 (95% UI, 34 to 55) per 100,000 person-years in 2019. In China, the ASIR decreased from 1990 to 1997, and then increased and peaked in 2004, and subsequently decreased continuously to 31 (95% UI, 26 to 36) per 100,000 person-years in 2019 (Fig. 3a, Table 1). The ASIR in the DPRK showed a downward trend from 1990 to 2019. The greatest decrease in ASDR per 100,000 person-years was found in the Republic of Korea, from 52 (95% UI, 50 to 55) in 1990 to 14 (95% UI, 13 to 16) in 2019 (Fig. 3b, Table 1). The ASDR in Mongolia initially showed an increasing trend from 1990 to 1996 and then decreased to 46 (95% UI, 37 to 57) per 100,000 person-years in 2019. In Japan, the ASDR per 100,000 person-years declined prominently from 32 (95% UI, 31 to 33) in 1990 to 14 (95% UI, 12 to 15) in 2019. The ASDR in the DPRK decreased gradually from 1990 to 2019, while that in China initially decreased from 1990 to 1998, increased and peaked in 2004, and then decreased continuously to 22 (95% UI, 18 to 25) per 100,000 person-years in 2019 (Fig. 3b, Table 1).

### Time trends in the incidence of, and death due to, gastric cancer from 1990 to 2019

**Figure 4:**
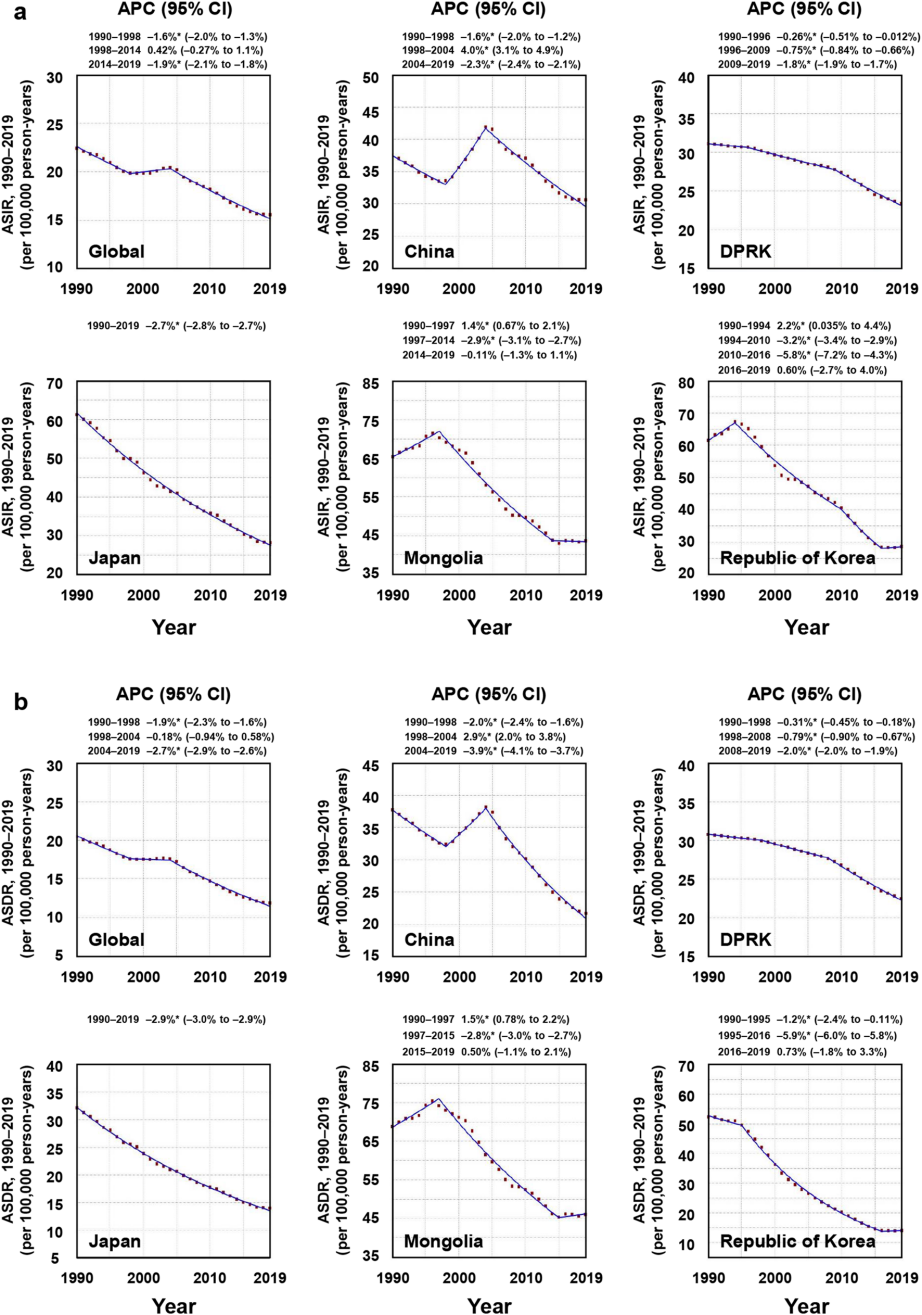
Estimates of APCs in ASIRs (**a**) and ASDRs (**b**) of gastric cancer worldwide and in five Asian countries from 1990 to 2019. ASDR, age-standardized death rate. ASIR, age-standardized incidence rate. DPRK, the Democratic People’s Republic of Korea. APC, estimated annual percentage change. Data estimated with 95% confidence intervals. *Trends between joinpoints significantly different from zero (*P* < 0.05).

The greatest falling trend in ASIR worldwide [APC, − 1.9% (95% CI, − 2.1 to − 1.8%)] was from 2014 to 2019 (Fig. 4a), and the greatest falling trend in ASDR worldwide [APC, − 2.7% (95% CI, − 2.9 to − 2.6%)] was from 2004 to 2019 (Fig. 4b).

For ASIR, the greatest rising trends were found in China from 1998 to 2004 [APC, 4.0% (95% CI, 3.1 to 4.9%)], the Republic of Korea from 1990 to 1994 [APC, 2.2% (95% CI, 0.035 to 4.4%)], and Mongolia from 1990 to 1997 [APC, 1.4% (95% CI, 0.67 to 2.1%)] (Fig. 4a). The greatest falling trends of ASIR were found in the Republic of Korea from 2010 to 2016 [APC, − 5.8% (95% CI, − 7.2 to − 4.3%)], Mongolia from 1997 to 2014 [APC, − 2.9% (95% CI, − 3.1 to − 2.7%)], and Japan from 1990 to 2019 [APC, − 2.7% (95% CI, − 2.8 to − 2.7%)] (Fig. 4a).

For ASDR, the greatest rising trends were found in China from 1998 to 2004 [APC, 2.9% (95% CI, 2.0 to 3.8%)], and Mongolia from 1990 to 1997 [APC, 1.5% (95% CI, 0.78 to 2.2%)] (Fig. 4b). The greatest falling trends of ASIR were found in the Republic of Korea from 1995 to 2016 [APC, − 5.9% (95% CI, − 6.0 to − 5.8%)], China from 2004 to 2019 [APC, − 3.9% (95% CI, − 4.1 to − 3.7%)], and Japan from 1990 to 2019 [APC, − 2.9% (95% CI, − 3.0 to − 2.9%)] (Fig. 4b).

### Incidence and death in relation to age and SDI

#### Effect of age

The incidence and death rates increased with age in all five Asian countries (Supplementary Fig. 1a, b). Globally, the incidence rate in 2019 increased with age, peaked at 85–89 years, and then dropped (Supplementary Fig. 2a). The same trend was seen in the Republic of Korea and China (Supplementary Fig. 2b, c). The incidence rate increased with age, peaked at 90–94 years, and then dropped in Mongolia and Japan (Supplementary Fig. 2d, e). In the DPRK, the incidence rate increased with age and dropped after 75–79 years of age (Supplementary Fig. 2f.). The death rate worldwide increased progressively with age and peaked at ≥ 95 years old (Supplementary Fig. 3a). Similar trends were found in the Republic of Korea, Japan, and Mongolia (Supplementary Fig. 3b, c, d). The death rate increased with age and dropped after 85–89 years of age in China (Supplementary Fig. 3e), and increased with age, fell after 85–89 years, and peaked at ≥ 95 years in the DPRK (Supplementary Fig. 3f.).

#### Effect of SDI

**Figure 5:**
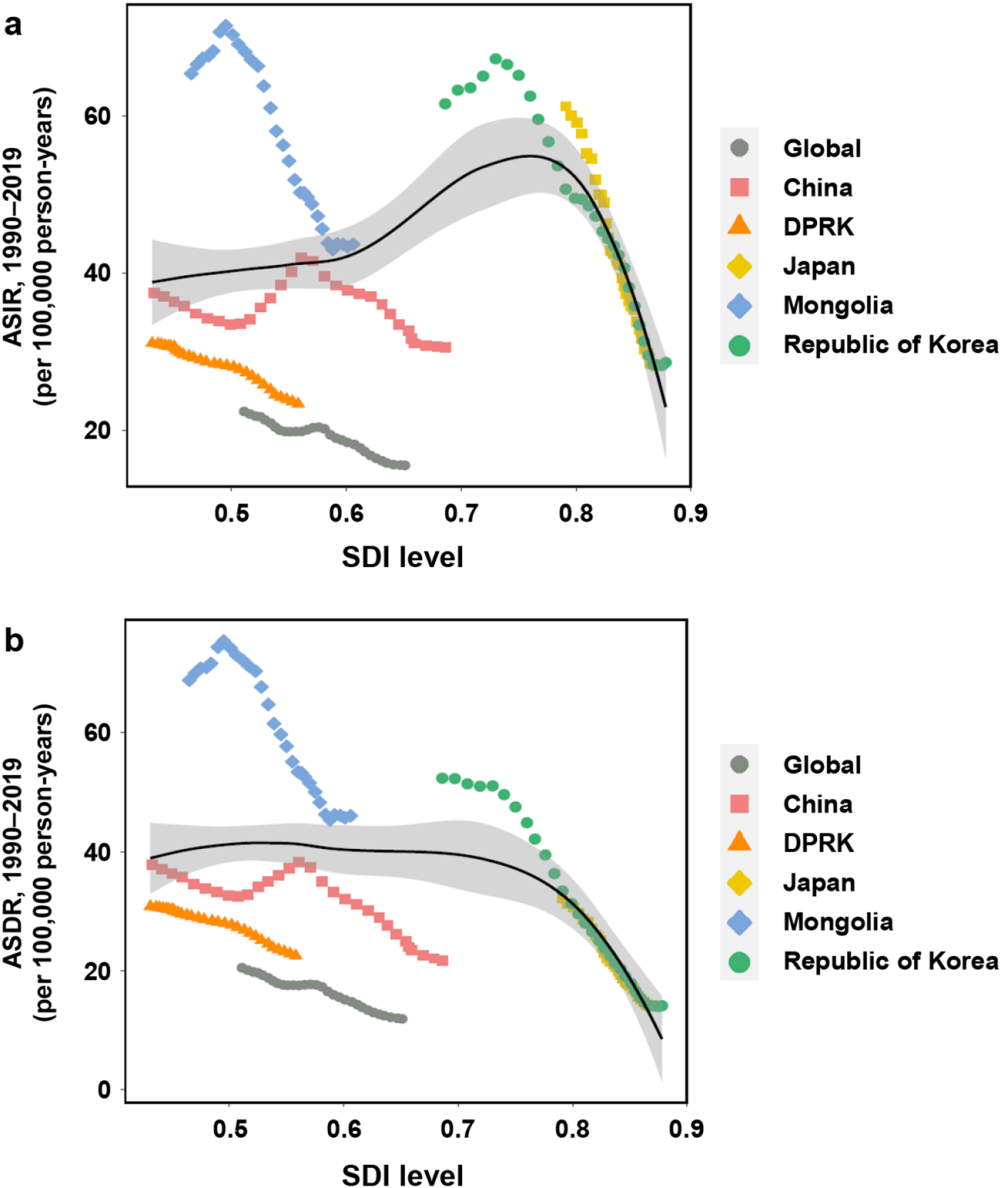
ASIRs (**a**) and ASDRs (**b**) of gastric cancer worldwide and in five Asian countries from 1990 to 2019 according to SDI. ASDR, age-standardized death rate. ASIR, age-standardized incidence rate. DPRK, the Democratic People’s Republic of Korea. SDI, sociodemographic index.

There were obvious differences in the effects of SDI among the five Asian countries (Fig. 5a). The ASIR in Japan decreased sharply whereas the ASIR in the DPRK decreased only slowly with increasing SDI. The ASIR in Mongolia initially increased, peaked at an SDI of 0.50 in 1996, and then decreased remarkably with increasing SDI. Similarly, the ASIR in the Republic of Korea initially increased, peaked at an SDI of 0.73 in 1994, and then decreased with subsequent increases in SDI. In China, the ASIR initially decreased from 1990 to 1997, increased and peaked at an SDI of 0.56 in 2004, and then decreased with further increases in SDI.

The ASDR in the Republic of Korea and Japan decreased sharply with increasing SDI, whereas the ASDR in the DPRK decreased more slowly (Fig. 5b). The ASDR in Mongolia initially increased, peaked at an SDI of 0.50 in 1996, decreased remarkably with increasing SDI until 2015, and finally remained stable irrespective of increasing SDI from 2016 to 2019. In China, the ASDR initially decreased from 1990 to 1998, increased and peaked at an SDI of 0.56 in 2004, and finally decreased with increasing SDI from 2004 to 2019.

#### Possible risk factors of gastric cancer

**Figure 6:**
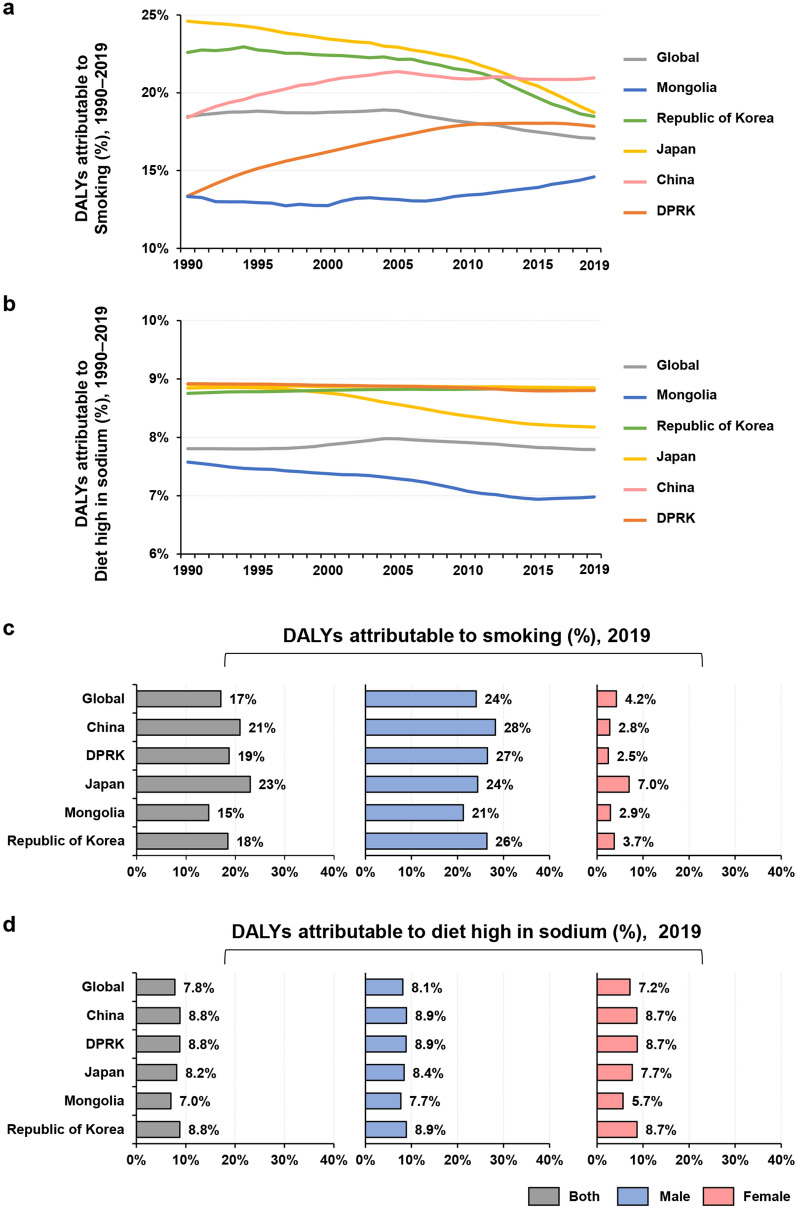
Trends of gastric cancer age-standardized DALYs attributable to smoking (**a**) and a high-sodium diet (**b**) from 1990 to 2019 according to sex. Proportions of age-standardized DALYs of gastric cancer attributable to smoking (**c**) and a high-sodium diet (**d**) worldwide and in five Asian countries in 2019. DALYs, disability-adjusted life-years. DPRK, the Democratic People’s Republic of Korea.

Figure 6 shows the global trends and proportions of age-standardized DALYs attributable to a high-sodium diet and smoking. The trends of age-standardized DALYs attributable to smoking from 1990 to 2019 varied among the five Asian countries. Japan and the Republic of Korea exhibited downward trends, whereas China, the DPRK, and Mongolia showed upward trends in age-standardized DALYs attributable to smoking (Fig. 6a). Regarding the age-standardized DALYs attributable to a high-sodium diet, the trends in the Republic of Korea, China, and DPRK remained stable, while Japan and Mongolia showed downward trends from 1990 to 2019 (Fig. 6b).

In 2019, the proportion of age-standardized DALYs attributable to smoking was highest in Japan [23% (95% UI, 19 to 28%)], followed by China [21% (95% UI, 17 to 25%)], the DPRK [19% (95% UI, 15 to 22%)], the Republic of Korea [19% (95% UI, 15 to 22%)], and Mongolia [15% (95% UI, 11 to 18%)] (Fig. 6c). The proportions of age-standardized DALYs were higher among males than females in all five countries: China [males 28% (95% UI, 23 to 33%); females 2.8% (95% UI, 2.0 to 3.6%)], Japan [males 24% (95% UI, 20 to 29%); females 7.0% (95% UI, 5.2 to 8.7%)], Mongolia [males 21% (95% UI, 16 to 27%); females 2.9% (95% UI, 1.7 to 4.4%)], the DPRK [males 27% (95% UI, 21 to 31%); females 2.5% (95% UI, 1.5 to 3.6%)], and the Republic of Korea [males 26% (95% UI, 22 to 31%); females 3.7% (95% UI, 2.7 to 4.8%)] (Fig. 6c).

The proportions of age-standardized DALYs attributable to a high-sodium diet differed slightly among countries and sexes in 2019. China [8.8% (95% UI, 0.21 to 33%)], the DPRK [8.8% (95% UI, 0.21 to 33%)], the Republic of Korea [8.8% (95% UI, 0.21 to 33%)], and Japan [8.2% (95% UI, 0.21 to 32%)] had higher proportions of age-standardized DALYs attributable to a high-sodium diet compared with the global level [7.8% (95% UI, 0.2 to 31%)], whereas Mongolia had a lower proportion [7.0% (95% UI, 0.22 to 30%)] than the global level (Fig. 6d). For males, the proportions of age-standardized DALYs associated with a high-sodium diet in China [8.9% (95% UI, 0.21 to 33%)], the Republic of Korea [8.9% (95% UI, 0.21 to 33%)], and the DPRK [8.9% (95% UI, 0.21 to 33%)] were higher than the global level [8.1% (95% UI, 0.22 to 32%)], while that in Mongolia [7.7% (95% UI, 0.21 to 31%)] was lower than the global level. For females, the highest proportions of age-standardized DALYs attributable to a high-sodium diet were found in China [8.7% (95% UI, 0.21 to 33%)], the Republic of Korea [8.7% (95% UI, 0.21 to 33%)] and the DPRK [8.7% (95% UI, 0.21 to 33%)], whereas the lowest were in Mongolia [5.7% (95% UI, 0.22 to 27%)] (Fig. 6d).

## Discussion

Gastric cancer is becoming a global health challenge with substantial morbidity and mortality, in Asia, particularly China, accounting for a large proportion of the global burden of gastric cancer^16^. Here, we carried out a comprehensive and systematic study to reveal the current burdens and trends in the incidence, death, and possible risk factors of gastric cancer in five Asian countries.

The most recent GLOBOCAN report^1^ estimated a total of 1,089,103 incident cases and 768,793 deaths due to gastric cancer in 2020. We estimated a total of 1,269,806 cases and 957,185 deaths in 2019. We found that the incidence and death in China and Mongolia were higher than those in Japan and the Republic of Korea. The reasons for this difference are not clear, but there are several possible explanations. First, malnutrition might lead to higher incidence and death rates in countries with a low SDI^17^. Second, a lack of early-screening awareness results in low detection rates of early cancers and thus affects the prognosis and burden of cancer patients in countries with a low SDI^18,19^. Most cases in Japan and the Republic of Korea were detected by early screening and these cases with a better prognosis thus accounted for larger proportions of cases overall in these two countries. Third, the availability and accessibility of health care may differ among the countries^20^. Finally, the application of new anticancer drugs and therapeutic strategies may lag behind in countries with a low SDI^21^. Moreover, we found that men had higher incidence and death rates of gastric cancer than women. These discrepancies could be attributed to differences in lifestyle (men are more likely to be culturally influenced to take up drinking and smoking than women), environmental or occupational exposure, and physiological differences^22–25^.

We also investigated the heterogeneous trends and changes in ASIR and ASDR, and compared the differences among the five Asian countries. From 1990 to 2019, the global ASIR and ASDR of gastric cancer gradually decreased, with substantial national heterogeneity. As with the national level, all five Asian countries showed percentage decreases in ASIR and ASDR between 1990 and 2019. One potential explanation for these decreases is that the early detection of *H. pylori* infection and the effective use of antibiotics are vital to prevent and control gastric cancer. Although we were unable to evaluate the role of *H. pylori* infection in the gastric cancer burden in the present study, most of the risk reduction associated with improved socio-economic status is thought to stem from reduced rates of *H. pylori* infection^26^. It is clear that the decreasing trends in gastric cancer occurrence parallel the decline in *H. pylori* infections in both Eastern and Western populations^27,28^.

The most significant decreasing trend of ASIR from 1990 to 2019 occurred in Japan and the most significant decreasing trend of ASDR were found in the Republic of Korea. However, the trends of ASIR and ASDR in China initially decreased from 1990 to 1997, increased to a peak in 2004, and then continuously decreased to 2019. These findings reflect the huge differences in cancer prevention and management among the five Asian countries. Japan and the Republic of Korea have implemented nationwide population-based screening programs for gastric cancer for several decades, and the observed reductions in death rates support the benefits of screening interventions^29^. It has been suggested that the trends of ASIR and ASDR in China for the period of 1990–2004 are associated with the development of cancer registration. The National Central Cancer Registry of China was founded in 2002 to act as a national bureau for the management of cancer registration^30^. Futhermore, the trends and changes of ASIR and ASDR in China for the period of 2004–2019 may reflect the introduction of screening tests, leading to increased detection and thus an apparently increased incidence of gastric cancer. The Chinese government has only implemented cancer screening based on populations in high-risk areas for gastric cancer since 2006^21^. Moreover, the increasing awareness of early cancer screening in China may partly explain the decline in ASDR of gastric cancer after 2006.

The variations in ASIR and ASDR of gastric cancer across the five Asian countries may also be explained by heterogeneity in the prevalence of risk factors. Our results suggest that lifestyle factors, particularly a high-sodium diet and smoking among males, play important roles in the gastric cancer burden. Both of these are also risk factors for other non-communicable diseases, and minimizing exposure to these factors is generally recommended in guidelines for a healthy lifestyle^31^. The World Health Organization recommends a maximum salt intake of 5 g/day^32^. The proposed mechanisms by which salt can cause gastric cancer include via direct damage to the gastric mucosa leading to hyperplasia of the gastric pit epithelium, with increased potential for mutations^33^. A high sodium intake has also been reported to potentially alter the viscosity of the protective mucous barrier and increase colonization by *H. pylori*, as a recognized risk factor for gastric cancer^34,35^. The current results highlight the importance of improving diet through public health interventions. Efforts to reduce salt intake in the Japanese diet began around 1965 and have continued to the present day, and might thus also be a reason for the decrease in the incidence of gastric cancer in Japan. Therefore, reducing high-salt foods in the diet is one of the proposed ways of tackling the gastric cancer problem in high-risk Asian countries^36^.

Smoking has historically been more common among males than females^7^, which may help to explain the higher burden of gastric cancer among males. Our study verified that the DALY rate attributable to smoking was higher among males than females. Tobacco products contain several carcinogens that have been linked to gastric cancer in humans^37^, and several potential mechanisms could explain the association between cigarette smoking and gastric cancer. Smoking-related DNA adducts that can bind to gastric mucosa DNA have been found in gastric cancers in smokers^38^. Cigarette smoking has also been associated with increased risks of dysplasia and intestinal metaplasia, which are precursor lesions of gastric cancer^39^. In addition, N-nitroso-compounds are present in cigarette smoke and may be involved in gastric carcinogenesis^40^.

A large study including 470,168 participants found that alcohol consumption (≥ 5 alcoholic drinks daily) was also associated with an increased risk of gastric cancer in the United States (odds ratio 3.13, 95% confidence interval 1.15 to 8.64)^41^, while another study with 54,682 participants also found that alcohol consumption was associated with an increased risk of gastric cancer among Japanese men, rather than women, regardless of the anatomical subsite of the cancer^42^. Heavy (≥ 7 times per week) and binge (≥ 55 g alcohol intake per occasion) alcohol consumption showed 3.48-fold and 3.27-fold higher risks, respectively, in subjects not previously infected with *H. pylori*, compared with non-drinkers^43^. Smoked and salted food were also associated with the risk of gastric cancer. A history of consumption of smoked meat was found in 77.8% of gastric cancer patients in India^44^, and salted fish was associated with an elevated risk of gastric cancer (highest intake vs. lowest intake, 1.17 times)^45^. The risk of gastric cancer was thus increased by 12% per 5 g/day increment of dietary salt intake^35^.

In the present study, we used SDI to reflect socioeconomic status. The ASIR and ASDR of gastric cancer in Japan showed remarkable and continuous decrease in line with increases in SDI from 1990 to 2019. It may reflect the fact that with increases in SDI mean that countries have more manpower and financial resources to invest in the early detection of gastric cancer, thus improving treatment outcomes of this disease. Meanwhile, gastric cancer patients need effective multimodal therapy including surgery, radiotherapy, chemotherapy, targeted therapy, and other therapies to achieve a better prognosis^46^, which can be facilitated by an increase in SDI. In our analysis, the epidemiologic profiles of the gastric cancer burden showed large heterogeneities. Investments in cancer prevention and treatment need to recognize the interdependence between socio-economic status and health. More efforts are needed to reduce these health inequalities and ensure the balanced development of health services in all countries.

Globally, the age-specific rate for the incidence of gastric cancer increased with age and peaked at 85–89 years. The age-specific rate for global death also increased progressively with age and peaked at ≥ 95 years. These results suggest that measures are needed to target very elderly adults in order to reduce mortality due to gastric cancer. However, it remains a major challenge for the management of elderly people (≥ 75 years), considering the higher risk of comorbidities, decreased physiological and cognitive functions^47^, making it difficult for the early detection among them. Higher early detection rate of gastric cancer will contribute to the mortality reduction for the elderly people, and the positive results of *H. pylori* antibody tests followed by endoscopy will increase this early detection rate, thereby improve the survival rates for elderly people. Therefore, it will be clinically important for the national-wide screening of *H. pylori* and followed by endoscopy, if necessary.

Efforts should thus be made to reduce the high burden of gastric cancer and its possible risk factors in these Asian countries. Nationwide screening in Japan and the Republic of Korea can facilitate the early detection of gastric cancer. Nationwide endoscopic screening of gastric cancer should also be provided and covered by national health insurance in the other three countries, followed by *H. pylori* eradiation if necessary. The application of national guidelines for the early detection of gastric cancer and *H. pylori* eradiation is therefore important. Regarding lifestyles and dietary factors, the use of serving chopsticks is strongly recommended, and efforts should be made to control smoking in public places; furthermore, heavy alcohol drinking and the intake of smoked and salty food should be decreased in daily life.

The current study still has limitations. First, it is difficult to distinguish cardia gastric cancer from non-cardia gastric cancer, which is correlated with *H. pylori* infection^5^. Cardia gastric cancer occurred more frequently in low-risk population^48^. It remains difficult to compare the burden and trends of cardia and non-cardia gastric cancer among different populations^49^. Second, it is not available to obtain data for the possible risk factors of gastric cancer, such as *H. pylori*. Third, the molecular subtypes of gastric cancer are also important, but such data is still lacking^16^. Finally, the current study may be partly limited by the low availability and quality of data in low SDI countries.

In conclusion, there are substantial variations in the incidence and death of gastric cancer in the five Asian countries. Beyond the health benefits associated with general improvements in socio-economic status, specific local strategies are needed to further reduce the number of cases and deaths due to gastric cancer. The main findings of this study may be helpful for the policymakers to make better public decisions and allocate appropriate resources.

## Supplementary Information

Supplementary Information 1.

Supplementary Information 2.

Supplementary Information 3.

Supplementary Information 4.

## Data Availability

The data generated and analyzed in this study are available from the Global Health Data Exchange query tool (http://ghdx.healthdata.org/gbd-results-tool). The data that support the main findings of this study are also available from the corresponding author (Chun-Dong Zhang) upon reasonable request.
