# Supplementary figures and images for: The burden and trend of gastric cancer and possible risk factors in five Asian countries from 1990 to 2019
Source: Sci Rep. 2022 Apr 8;12:5980. doi: 10.1038/s41598-022-10014-4 (PMC8993926; doi:10.1038/s41598-022-10014-4)

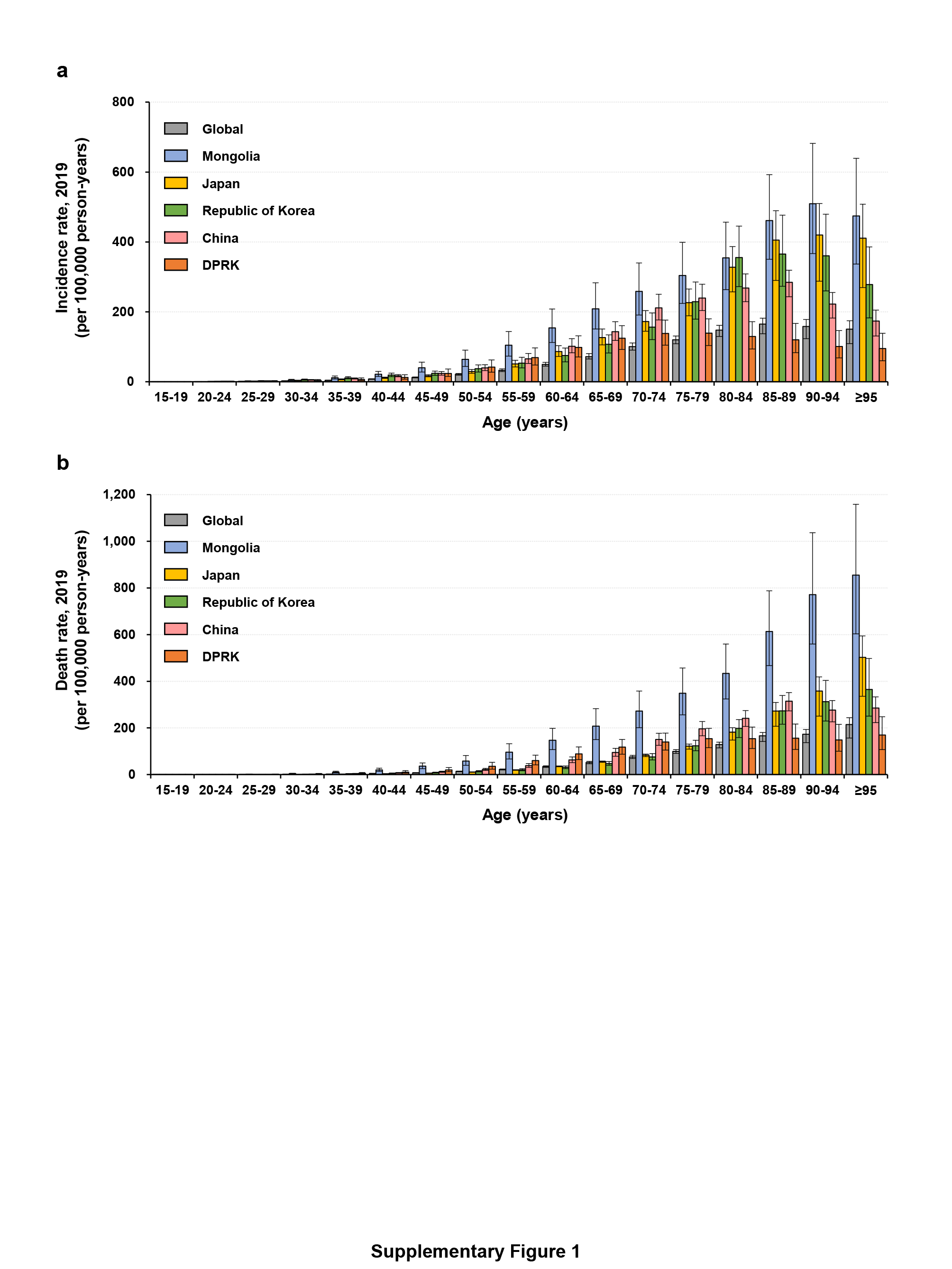

Supplement: Supplementary file 1 — Supplementary Information 1. [file 41598_2022_10014_MOESM1_ESM.tif]

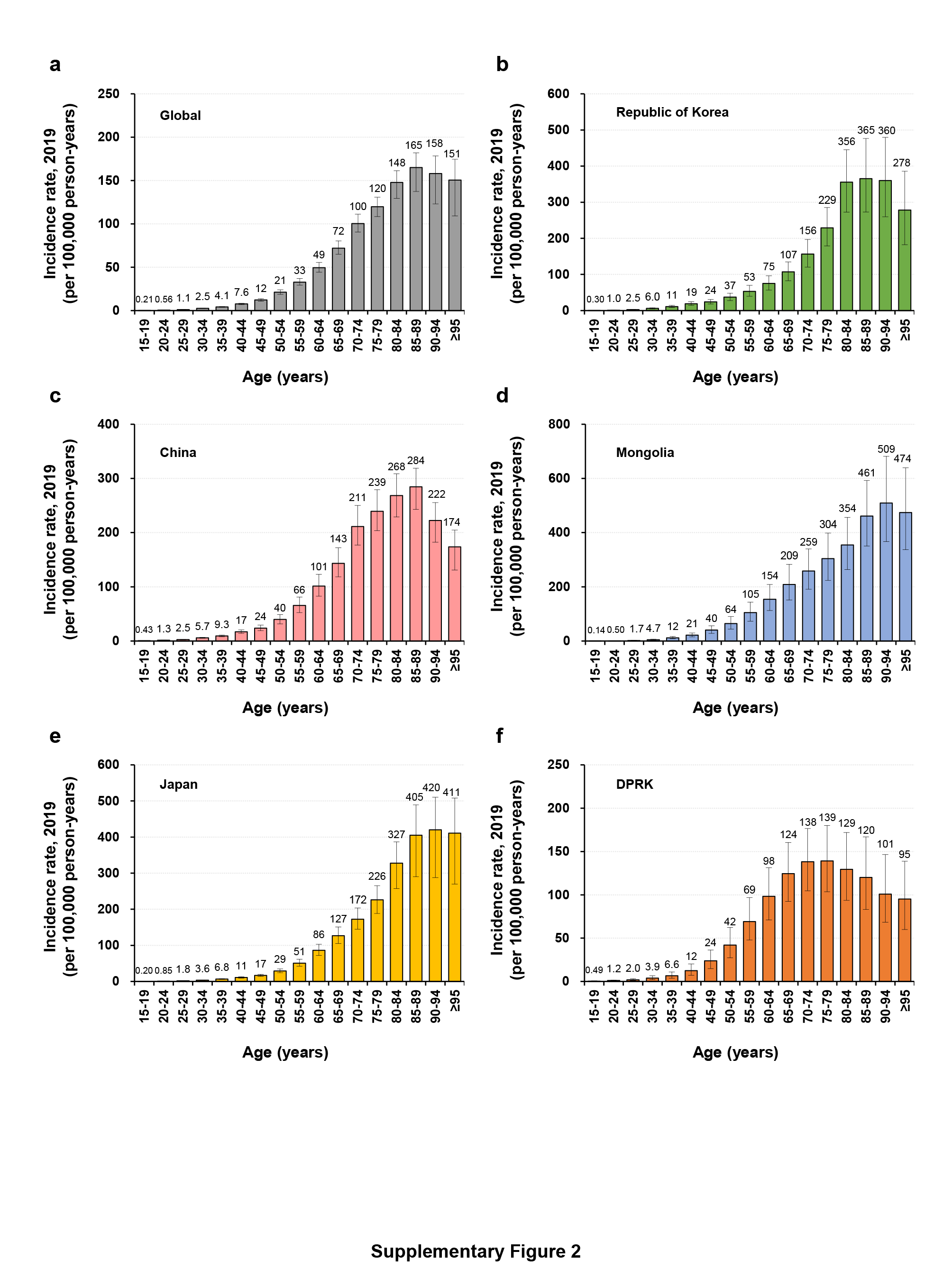

Supplement: Supplementary file 2 — Supplementary Information 2. [file 41598_2022_10014_MOESM2_ESM.tif]

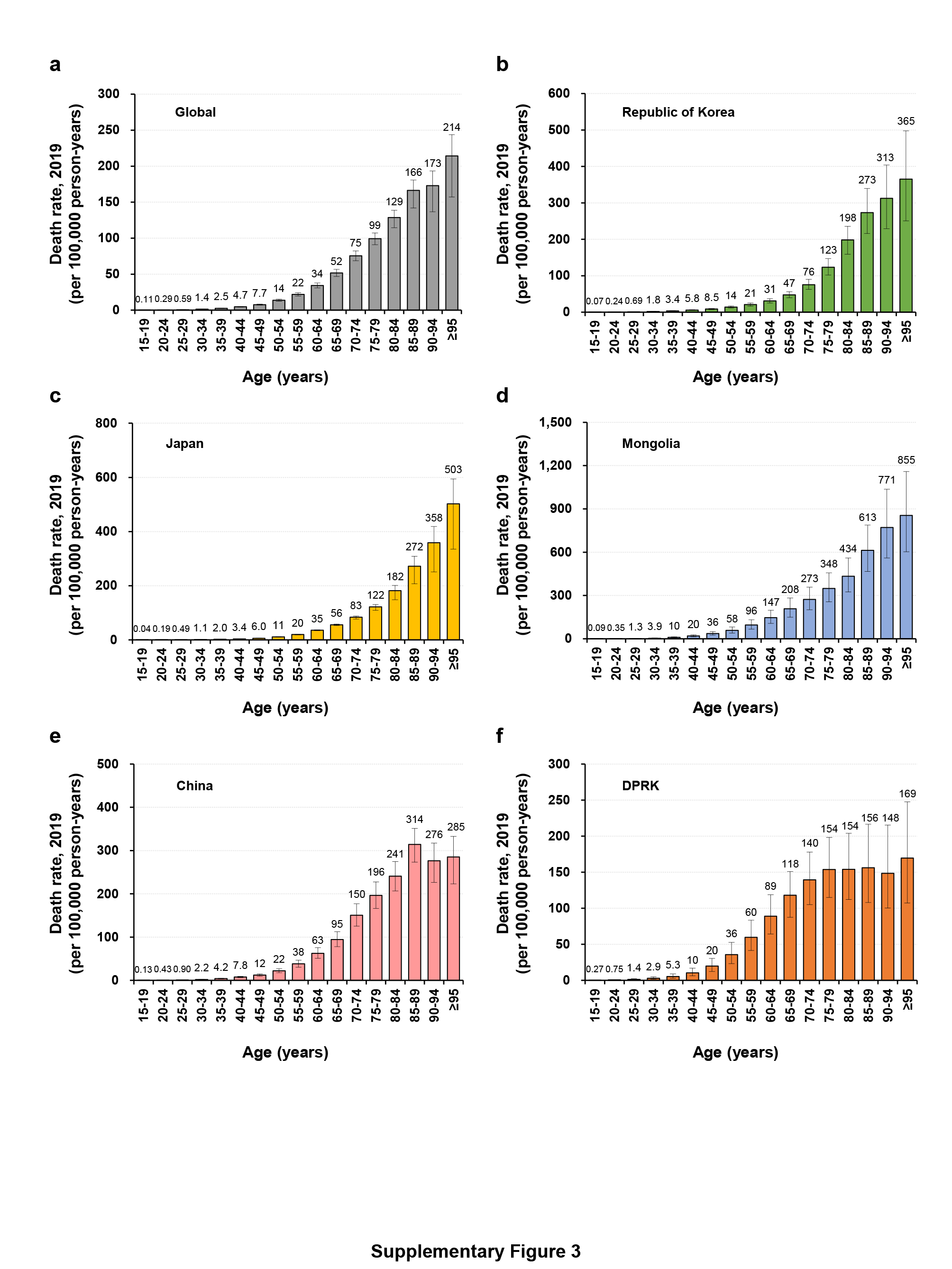

Supplement: Supplementary file 3 — Supplementary Information 3. [file 41598_2022_10014_MOESM3_ESM.tif]
