# Supplementary material for: The burden and trend of gastric cancer and possible risk factors in five Asian countries from 1990 to 2019
Source: Sci Rep. 2022 Apr 8;12:5980. doi: 10.1038/s41598-022-10014-4 (PMC8993926; doi:10.1038/s41598-022-10014-4)
Supplement: Supplementary file 4 — Supplementary Information 4. [file 41598_2022_10014_MOESM4_ESM.docx]

**Supplementary Figure legends for online only**

**Supplementary Figure legends**

**Supplementary Figure 1** Incidence rates (a) and death rates (b) of gastric cancer in the world and five Asian countries in 2019 according to age. DPRK, the Democratic People’s Republic of Korea.

**Supplementary Figure 2** Incidence rates of gastric cancer in the world and in five Asian countries in 2019 according to age. (a) Global, (b) the Republic of Korea, (c) China, (d) Mongolia, (e) Japan, and (f) DPRK. DPRK, the Democratic People’s Republic of Korea.

**Supplementary Figure 3** Death rates of gastric cancer in the world and five Asian countries in 2019 according to age. (a) Global, (b) the Republic of Korea, (c) China, (d) Mongolia, (e) Japan, and (f) DPRK. DPRK, the Democratic People’s Republic of Korea.
